# Return to sport after arthroscopic meniscectomy on stable knees

**DOI:** 10.1186/2052-1847-5-23

**Published:** 2013-11-20

**Authors:** Sung-Gon Kim, Masashi Nagao, Koichi Kamata, Koichi Maeda, Masahiko Nozawa

**Affiliations:** 1 Department of Opthopaedic Surgery, Juntendo University Nerima Hospital, Tokyo, Japan

**Keywords:** Meniscus, Return to sports, Stable knee

## Abstract

**Background:**

Athletes suffering from any injuries want to know when they will be able to return sports activity. The period of return-to-sport after the arthroscopic meniscectomy is still unknown. The aim of this study is to investigate the period of the return-to-sport from surgery and the clinical symptoms after the meniscectomy on stable knees.

**Methods:**

Fifty-six athletes who underwent the arthroscopic meniscectomy were evaluated. The patients with an average age of 26.7 years (range, 13–67) comprised 45 men and 11 women, 16 medial meniscus and 40 lateral meniscus injuries. The average of the follow-up period was 9.2 months. The parameter examined were age, the injured side of meniscus (medial or lateral), articular cartilage status, amount of resection, and sports activity level.

**Results:**

The mean period was 54 days in young group, and was 89 days in old group (*p* = 0.0013). The period was 79 days in medial meniscus (MM) injured group, and was 61 days in lateral meniscus (LM) group (*p* = 0.017). There was a significant difference among the groups in activity levels and in amount of resection. Pain and/or effusion in the knee after the return-to-sport were found 22% of the MM group and 53% in the LM group.

**Conclusions:**

The period of the return-to-sport was shorter in young age, high activity and large amount of resection group. Although athletes in LM group can return to sports earlier than those in MM group, more than half of athletes have pain or effusion at the time of return-to sport.

## Background

The meniscus of the knee is commonly injured during sports activity. The meniscus is known to play important roles in load share, shock absorption, lubrication, and knee stability [1-3]. Previous studies reported that meniscal excision leads to an increased risk of articular degeneration [4-6]. So meniscal tears should be considered for repair if possible [7-11]. But the meniscal repair takes longer rehabilitation period than the meniscectomy [12,13]. We have performed meniscectomy for the cases that meniscal tears are not repairable and occasionally patients desire early return to sports. Athletes suffering from injuries almost always want to return to sports activity as soon as possible, and also want to know when they will be able to return it. But the period of return-to-sport after the meniscectomy is still unknown. In addition, rehabilitation program has not been standardized after the surgery. The aim of this study is to investigate the period of the return-to-sport from surgery and the clinical symptoms at the time of return after the arthroscopic meniscectomy on stable knees. Our hypothesis was that the period of return-to-sport after the meniscectomy would differ by age, the side of meniscus, articular cartilage status, amount of resection volume and sports level.

## Methods

This study received the approval of the ethics committee of Juntendo University Nerima Hospital of our institution (No. 13–15). From April 2006 and December 2010, a total of 68 athletes underwent partial meniscectomy in our institution. Fifty-six (82.4%) patients who could be followed up until return-to-sport were eligible for this study. All patients had no cruciate ligament injuries or previous surgeries. Surgeries were performed arthroscopically under general anesthesia. Full weight bearing, range of motion exercise, quadriceps setting and straight leg raising exercise were allowed from the first postoperative day. Closed kinetic chain exercise was permitted if knees had no pain. Running was commenced at 4 weeks after surgery, progressing to sports specific activities. After that the full sports activity was resumed, if possible.

The patients with an average age of 26.7 years (range, 13–67) comprised 45 men and 11 women, 16 medial meniscus and 40 lateral meniscus (include 8 discoid meniscus) injuries. There were 5 athletes over 50 years old, but they were included because their Tegner score were 6. The average of the follow-up period was 9.2 months. The period of the return-to-sport from surgery and clinical symptom at the time of return was investigated. Athletes were followed up every month after surgery, and the dates they returned to sports were recorded. The definition of the return-to-sport is preinjury sports participation whether athletes have pain and effusion on the knees or they degrade their performance of the sports.

The parameter examined were age, the injured side of meniscus (medial or lateral), articular cartilage status, amount of resection, and sports activity level. The age at surgery was divided into young (<30; n = 36) and old (≥30; n = 20) group. At the time of surgery, any articular cartilage seen was rated according to the grading system described by Noyes and Stabler, and grade 2 and/or more was defined as damaged cartilage. The amount of resection was divided into small (<1/3), moderate (≥1/3, <2/3), and large (≥2/3) group. The preinjury sports activity level was used to classify the patients into elite (national elite, professional or division 1 college athlete, average Tegner activity score 9.3), competition (competitive sports excepting “elite”, Tegner 8.3), and recreation (sports at least once a week, Tegner 6.6).

Statistical analyses were performed using GraphPad Prism 5 software (GraphPad Software, Inc, San Diego, CA, USA). The results were analyzed using Mann–Whitney U-test for comparing two groups, and Kruskal-Wallis test for three groups. Chi-square test or Fisher’s exact test was used in the case of categorical variables. *P* values less than 0.05 were considered to indicate statistical significance.

## Results

The common sports performed by the athletes before meniscal injury were soccer (15 cases), basketball (10), baseball (7), volleyball (5), and rugby (4). Twenty-one percent of patients (12 cases) were elite group, 41% (23 cases) were competition group, and 38% (21 cases) were recreation group.

**Figure 1:**
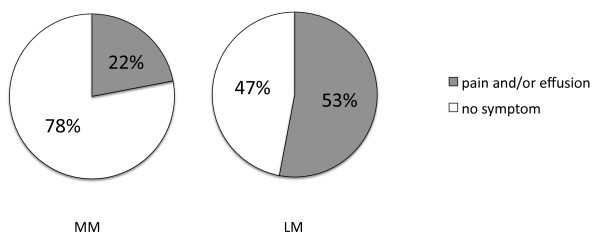
Pain and/or effusion in the knee after the return-to-sport.

The periods of return-to-sport from surgery were summarized in Table 1. The mean period was 54 days in young group, and was 89 days in old group (*p* = 0.0013). The period was 79 days in medial meniscus (MM) injured group, and was 61 days in lateral meniscus (LM) group (*p* = 0.017). There was no significant difference between the groups in cartilage surface. There was a significant difference among the groups in amount of resection (p = 0.0155, between small group and large group). But there was no significant difference between the site and pattern of tear (anterior, mid or posterior horn, longitudinal, radial, horizontal or complex tear). The mean period was 54 days in elite group and was 53 days in competition group, and was 88 days in recreation group. There was a significant difference among activity levels (*p* = 0.0036, between recreation group and elite and competition groups), and was also a significant difference among the groups in age (p < 0.0001, between recreation group and elite and competition groups). Pain and/or effusion in the knee after the return-to-sport were found 22% (4/16) of the MM group and 53% (21/40) in the LM group (Figure 1). In small group, 7 cases had pain and/or effusion and 10 cases had no symptom, and in moderate group, 11 and 11 cases, and in large group, 7 and 10 cases, respectively. There was no significant tendency between the amount of resection and pain and/or effusion. Quadriceps muscle training and icing after the sports were instructed for pain and effusion, continuing the sports activity. But 3 athletes in the LM group underwent a second-look arthroscopic meniscectomy after the return-to-sport. The lateral menisci of the 3 athletes were not able to be clearly detected tears in MRI before the reoperation. Degenerative changes in the remaining menisci were found arthroscopically. All of 3 athletes returned to previous sports without pain after the resection of degenerative portion.

**Table 1:** Summary of period of return-to-sport from surgery

	**Number**	**Period of return-to-sport mean (95% CI), days**	***p *** **value**
Age			
Young (<30)	36	54 (46–61)	*p* = 0.0013
Old (≥30)	20	89 (69–109)	
Injured meniscus			
MM	16	79 (63–95)	*p* = 0.017
LM	40	61 (50–73)	
Cartilage surface			
Smooth	43	62 (53–71)	n. s.
Damaged	13	80 (51–109)	
Amount of resection			
Small (<1/3)	17	79 (65–93)	*p* = 0.0155
Moderate (≥1/3, <2/3)	22	65 (47–84)	
Large (≥2/3)	17	54 (39–69)	
Activity level			
Elite	12	54 (37–70)	*p* = 0.0036
Competition	23	53 (43–63)	
Recreation	21	88 (69–107)	

n. s., not significant.

CI, confidence interval.

## Discussion

Many studies of the clinical outcome after the meniscectomy have been reported [5,12,14-18]. To our knowledge, however, only one study focusing on factors regarding the period of the return-to-sport after surgery has been published. That study, by Osti et al. [19], investigated the results after lateral meniscectomy. They reported that the mean period of return-to-sport from surgery was 41 days in athletes who had isolated, longitudinal tears of the lateral meniscus. Athletes with complex meniscal tears and tears associated with cartilaginous lesions required averaging 64 and 78 days, respectively. These results are similar to our results that showed that a mean of 61 days needed for all athletes after lateral meniscectomy. Associated cartilaginous injury in LM group, the periods were 55 days in no damaged cartilage group and 82 days in damaged group.

In our study, the period of return-to-sport from surgery in LM injured group was significantly shorter than that of MM injured group. However, pain and/or effusion in the knee after the return-to-sport occurred more frequently in LM group, and three athletes in LM group obliged to undergo repeat surgeries. Many previous studies reported that clinical results and radiologic changes were worse after lateral than medial meniscectomy [15,18,20]. The lateral meniscus is particular importance in young athletes because of the convexity of the femoral condyle against the convexity of lateral tibial plateau and more relative rotational movement due to femoral roll-back occurring mainly on the lateral side [6,13]. Lateral meniscal injuries were found to be more frequent than medial injury in our series. Similarly, in the National Basketball Association players, lateral meniscal tears were more common and prevalent up to the age of 30 years [21].

According to Matthews and St-Pierre [22], the quadriceps recovered to preoperative values by 4 to 6 weeks after arthroscopic meniscectomy. So in our postoperative protocol, running commenced at 4 weeks after surgery. If running starts immediately after operation, athletes may return much earlier. However, current study showed the high incidence of pain and effusion after the lateral meniscectomy, so the postoperative program should be considered carefully.

In this study, the high activity group returned to sports significantly earlier than the recreational level. Several factors may affect this result such as motivation for return-to-sport, muscle strength, time and environment for rehabilitation. Age also may be important factor of return-to-sport. Young group returned to sports earlier than older group. The high activity group contained a large number of young age athletes, so they probably interacted with each other.

Considering the importance of the meniscal function, minimum necessary amount of meniscal tissue should be resected in the partial meniscectomy. The large amount of meniscal resection has the risk for worsening knee function and radiological findings at long-term follow-up. Hede et al. [16] reported that a higher level of knee function was achieved after partial meniscectomy than after total meniscectomy because partial meniscectomy produced less joint instability. Englund et al. [17] showed that patients with degenerative tears scored significantly worse on the knee-specific outcomes after subtotal meniscectomy than after partial meniscectomy. The authors, however, described that the results didn’t apply to traumatic tears. In our results, athletes who underwent large amount of resection returned significantly earlier than those with small amount of resection, but the reason cannot be found in this study. Even if the meniscectomy is performed partially, large amount of resection often loses the hoop effect. Further research and long-term follow-up are necessary to evaluate the biomechanics and the clinical outcomes regarding the amount of resection.

There are some limitations for this study. The sample size was small, and therefore we could not investigate the effects of tear type and location. Although age, activity level and the injured side of meniscus (medial or lateral) are affiliated with each other, we could not analyze the data among matched subgroups. Besides, the follow-up periods are short, so we did not evaluate the results with widely used knee score. But the aim of this study was to investigate the period of the return-to-sport from surgery and the influential factors. Further studies are necessary to evaluate the long-term clinical symptom, especially after lateral meniscectomy because pain and/or effusion after the return-to-sport occurred more frequently in LM group.

## Conclusion

The factors affecting the period of the return-to-sport from surgery was investigated in this study. The period of the return-to-sport was shorter in young age, high activity and large amount of resection group. Although athletes in LM group can return to sports earlier than those in MM group, more than half of athletes have pain or effusion in the knee at the time of return-to sport.

## Competing interests

The authors declare that they have no competing interests.

## Authors’ contributions

SK designed this study and wrote the manuscript. MN assisted in writing the manuscript and the statistical analysis. KK and KM assisted in acquisition of data. MN contributed to analysis and interpretation of data. All authors read and approved the final manuscript.

## Pre-publication history

The pre-publication history for this paper can be accessed here:

http://www.biomedcentral.com/2052-1847/5/23/prepub
